# Comparative Analysis of Fluorinated Anions for Polypyrrole Linear Actuator Electrolytes

**DOI:** 10.3390/polym11050849

**Published:** 2019-05-10

**Authors:** Nguyen Quang Khuyen, Zane Zondaka, Madis Harjo, Janno Torop, Tarmo Tamm, Rudolf Kiefer

**Affiliations:** 1 Conducting Polymers in Composites and Applications Research Group, Faculty of Applied Sciences, Ton Duc Thang University, Ho Chi Minh City, Vietnam; nguyenquangkhuyen@tdtu.edu.vn; 2 Intelligent Materials and Systems Lab, Faculty of Science and Technology, University of Tartu, Nooruse 1, 50411 Tartu, Estonia; zane.zondaka@ut.ee (Z.Z.); madis.harjo@gmail.com (M.H.); jantor@ut.ee (J.T.); tarmo.tamm@ut.ee (T.T.)

**Keywords:** PPy/DBS, linear actuators, isotonic ECMD, four different electrolytes, actuation direction

## Abstract

Either as salts or room temperature ionic liquids, fluorinated anion-based electrolytes have been a common choice for ionic electroactive polymer actuators, both linear and bending. In the present work, propylene carbonate solutions of four electrolytes of the three hugely popular anions—triflouromethanesulfonate, bis(trifluoromethane)sulfonimide, and hexafluorophosphate were compared and evaluated in polypyrrole linear actuators. The actuation direction, the characteristics—performance relations influence the behavior of the actuators. Isotonic Electro-chemo-mechanical deformation (ECMD) measurements were performed to study the response of the PPy/DBS samples. The highest strain for pristine PPy/DBS linear actuators was found in range of 21% for LiTFSI, while TBAPF_6_ had the least cation involvement, suggesting the potential for application in durable and controllable actuators. Interesting cation effects on the actuation of the same anions (CF_3_SO_3_^−^) were also observed.

## 1. Introduction

Many applications have been proposed for conducting polymer actuators [1], also called “artificial muscles” [2], such as microactuators [3], soft robotics [4], biomedical devices [5], tissue engineering [6,7], sensors [8], and smart textiles [9]. Conducting polymer linear actuators have been investigated for a decade, initially focusing on strain optimization [10], later also on understanding the underlying actuation mechanism [11,12,13] brought along by the electrochemical redox reactions. In general, the electrochemically stimulated conformational relaxation model [14] describes the electrochemical reactions with the reversible volume changes of the polypyrrole (PPy) actuators most comprehensively at each stage: reduction-shrinking, reduction-compaction and oxidation-relaxation, and oxidation swelling [15,16]. The actuation of ionic actuators can be anion-driven [17], whereas at oxidation the positive charges generated in the PPy chains at (PPy^n+^) lead to counterions (anions, A^−^) and solvent (S) ingress, triggering volume change, described by Equation (1).

(1) $$\left(PPy^{0}\right)+n{\left(A^{-}\right)}_{sol}+m\left(S\right)⇄[\left(PPy^{n+}\right){\left(A^{-}\right)}_{n}\left(S{)}_{m}\right]+n\left(e^{-}\right)$$

Several works have demonstrated that anion-active PPy polymerized in propylene carbonate solutions of large(r) anions such as bis(trifluoromethane)sulfonimide (TFSI^−^) can achieve a high strain in a range of over 25% [18]. On the other hand, even larger dopant anions, such as dodecylbenzenesulfonate (DBS^−^) or phosphotungstinate [19], usually used during electropolymerization from aqueous solutions, end up as immobile in the PPy network. During reduction, their excess negative charge is balanced by the ingress of cations (C^+^) with solvent molecules (S) leading to expansion upon reduction, i.e., cation-driven actuators [17], described by Equation (2).

(2) $$\left(PPy^{0}\right){\left(DBS^{-}\right)}_{n}{\left(C^{+}\right)}_{n}{\left(S\right)}_{m}]⇄[\left(PPy^{n+}\right){\left(DBS^{-}\right)}_{n}\left(S{)}_{m}\right]+n\left(C^{+}\right)+m\left(S\right)+n\left(e^{-}\right)$$

To have control over the actuation direction and achieve high strains, as pure as possible anion or cation activity would be optimal, however, in most cases mixed ion incorporation takes place [20]. Other factors apart of the size of the (solvated) ions also play their role, like the solvent [15,21]. A typical PPy/DBS actuator is expected to be mainly cation-driven in hydrophilic solvents, but changed to anion driven if apolar aprotic solvents are applied [22,23,24]. Therefore, Equation (3) can be formulated to explain anion-driven actuation in PPy/DBS actuators [15], with reduction on the left side and oxidation on the right.

(3) $$[\left(PPy\right)\;{\left(DBS^{-}\right)}_{m}{\left(C^{+}\right)}_{m}\left(S{)}_{o}\right]+\;nA^{-}+\;pPC⇄[{\left(PPy\right)}^{n+}{\left(DBS^{-}\right)}_{m}{\left(C^{+}\right)}_{m}{\left(S\right)}_{\left(o+p\right)}\left(A^{-}{)}_{n}\right]+n{\left(e^{-}\right)}_{metal}$$

The immobile DBS^−^ anions in the PPy network are coupled with cations forming un-dissociated ion pairs [23] in aprotic solvents. Therefore, upon oxidation new locations in PPy (charged PPy^n+^) are filled by additional solvated anions (Equation (3)). 

The goal of this work was to investigate different electrolytes with fluorinated anions in propylene carbonate solutions applied for the linear actuation of PPy/DBS films. While tetrafluoroborate has fallen out of favour due to issues with stability leading to hydrolysis, other fluorinated anions have gained popularity in various applications, bis(trifluoromethane)sulfonimide and triflouromethanesulfonate in particular. Applications can be found in static charge dissipation [25] where flourninated anions applied to dope chemical PPy, and other applications can be found for fluorinated ionic liquids [26] in biomedical use. The main reason for why large fluorinated anions are applied in linear conducting polymer actuators relies on their large distribution of negative charge which is improving the anodic expansion [21].

Cyclic voltammetry and square wave potential step measurements were applied as the driving signals for actuating PPy/DBS films in different electrolytes. The actuation properties including their diffusion coefficients are compared, attempting to explain the differences and the roles of both cations and anions in the dominantly anion-active systems. SEM images and EDX spectroscopy were introduced to evaluate the changes taking place during actuation.

## 2. Material and Methods

### 2.1. Materials

Sodium dodecylbenzenesulfonate (NaDBS, technical grade) and ethylene glycol (EG, technical grade) were used for electropolymerization, and bis(trifluoromethane)sulfonimide lithium salt (LiTFSI, 99.95%), lithium triflouromethanesulfonate (LiCF_3_SO_3_, 99%), tetrabutylammonium triflouromethanesulfonate (TBACF_3_SO_3_, 99%), tetrabutylammonium hexafluorophosphate (TBAPF_6_) and propylene carbonate (PC, 99%) were applied as electrolytes in actuation studies and obtained from Sigma-Aldrich (Taufkirchen, Germany) and used as supplied. Pyrrole (Py, 98%, Sigma-Aldrich) was vacuum-distilled prior to use and stored at low temperature in the dark. Pyrrole was the monomer applied in the electropolymerization. Milli-Q+ water was used for making aqueous solutions. 

### 2.2. Electroformation of PPy/DBS Films 

The synthesis was carried out in a EG:Milli-Q (1:1) solution of 0.1 M pyrrole and 0.1 M NaDBS using. The galvanostatic electropolymerization was performed at 0.1 mA cm^−2^ (40.000s) in a two-electrode cell at −20 °C using a stainless-steel sheet working electrode (18 cm^2^) and a stainless-steel mesh counter electrode. The deposited PPy/DBS films were peeled off the stainless-steel working electrode, washed several times in Milli-Q to remove excess NaDBS and in ethanol to remove excess of the unreacted pyrrole monomer. The films were dried in an oven at 2 mbar at 60 °C for 24 h. The PPy/DBS films were stored in each studied electrolyte for 24 h before the measurements commenced.

### 2.3. Isotonic Electro-Chemo-Mechanical Deformation (ECMD) Measurements 

The PPy/DBS films were cut to strips of 1.2 cm length and 0.1 cm width. In a three electrode setup with platinum counter electrode, Ag/AgCl (3M KCl) wire reference electrode, the PPy/DBS films were fixed on the gold contact/electrode of a force sensor (TRI202PAD, Panlab) on the linear muscle analyzer setup [24]. The in-house ECMD measurement set-up had a movable force sensor giving an advantage over commercially available systems, where the force sensors are static. Before actuation, the mass required for displacing the force sensor by 1 µm was measured to determine the elastic modulus of the PPy/DBS films. The strain was determined from the length change of the films ε(%) = (*L*_1_ − *L*_0_/*L*_0_) × 100% (isotonic—constant force of 4.9 mN), where *L*_0_ is the original length of the film clamped between force sensor and the gold contact and L_1_ the new length of the film. The PPy/DBS films were operated in propylene carbonate solutions of different electrolytes (LiTFSI, LiCF_3_SO_3_, TBACF_3_SO_3_, and TBAPF_6_) at the same concentration of 0.2 M. The change in mass for 1 µm length change for PPy/DBS films in propylene carbonate solutions of LiTFSI was 29 mg/µm, in LiCF_3_SO_3_ 118 mg/µm, TBACF_3_SO_3_ 254 mg/µm, and for TBAPF_6_ 239 mg/µm. Cyclic voltammetry (CV) measurements with scan rate of 5 mVs^−1^, within voltage range of 1.0 V to −0.55 V were applied to drive the isotonic ECMD measurements. Square wave potential steps driving was also applied in frequency range of 0.0025 Hz to 0.1 Hz. For each measurement, three separate samples were used, and the mean values with standard deviation of the results are presented. 

### 2.4. Characterization of PPy/DBS Samples after Actuation 

After actuation in the four different electrolytes, the PPy/DBS samples were characterized by scanning electron microscopy (Helios NanoLab 600, FEI, Oregon, USA) and energy-dispersive X-ray spectroscopy (EDX) (Oxford Instruments with X-Max 50 mm^2^ detector). Before characterization, 5 min polarization at −0.55 V and +1.0 V was performed for the reduced and oxidized films, respectively. To evaluate the surface conductivity of the PPy/DBS films, an in-house 4-point probe was applied. Conductivity was calculated by Equation (4):

(4) $$\sigma_{e}=\frac{1}{\left(R*w\right)}$$

where σ_e_ is the electric conductivity, *R* is the surface resistivity (Ω/sq), and w is the material thickness.

## 3. Results and Discussion

**Figure 1:**
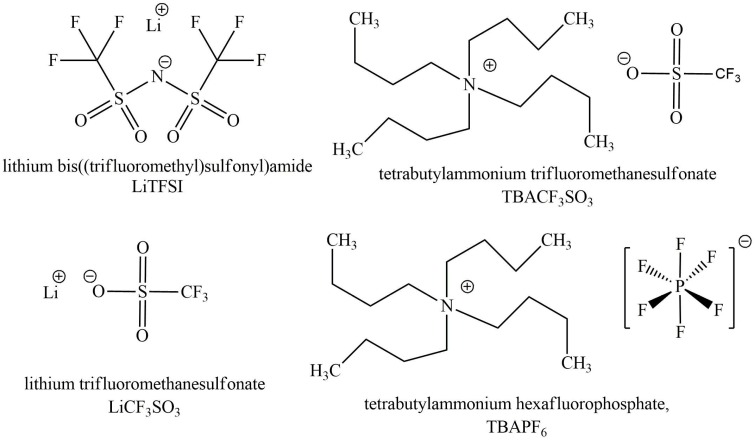
The structures of electrolytes LiTFSI, LiCF_3_SO_3_, TBACF_3_SO_3_, and TBAPF_6._

There are three main factors determining the mobility of ions in a conducting polymer film. These are the size of the ions, with and without the solvation shells in the particular solvent, and also possible (specific) interactions with the polymer network. Figure 1 shows the structure of the cations and anions of the electrolytes applied in the experiments. 

The small hydrophilic, high charge density Li^+^ ions applied before in the solvent exchange of PPy/DBS linear actuators [15,24] are here represented (Figure 1) by two electrolytes: LiTFSI and LiCF_3_SO_3_. The other cation—the more hydrophobic TBA^+^—is represented by TBACF_3_SO_3_ and TBAPF_6_ (Figure 1). Therefore, there are two cation pairs with one common and one uncommon anion. The overall behavior of the electrolytes is also influenced by the original dopant anion—DBS^−^. It is well known that DBS^−^ anions are large amphiphilic molecules [27], with their bulkiness and the aromatic nature responsible for being immobile in PPy network [28]. The more hydrophilic and higher charge density Li^+^ ions are supposed to attach closer and stronger to the sulfonate groups, while the hydrophobic and lower charge density TBA^+^ ions have less interaction and are more scattered around the DBS^−^ anions. The numeric values of some parameters influencing the behavior of the electrolytes applied in this work such as ion radius, single ion limited ionic conductivity λ_0_ [29], and solvation number in PC are presented in Table 1. 

There appears to be no commonly accepted figures for the solvation of the solvation numbers of the fluorinated anion in propylene carbonate. It has been discussed that the anion solvation relates to the hydrocarbon moieties (van der Waals force) of PC molecules, therefore the solvation must be very weak [32], while Li^+^ ions are highly solvated by the carbonate groups of PC molecules. As seen from Table 1, the ionic radius of Li^+^ is by far the smallest but the solvation number is the highest, while the highest ionic radius of TBA^+^ cations corresponds to negligible solvation [31]. In pure solution, the ion mobility is primarily controlled by its size [29].

### 3.1. Morphology

The SEM images (Figure S1) of PPy/DBS show that despite the remarkably different elastic moduli other characteristics, the morphology of the films actuated in different electrolytes have virtually no difference, all representing similar roughness with the typical cauliflower structure [33]. Therefore, the different properties and behavior of the electrolytes were not significantly transferred to the structure (at least of the surface) of the polymer network. 

The electronic conductivities of the PPy/DBS films operated in different electrolytes are presented in Table 2.

The swelling due to solvation has been studied before [21], and considered as relatively minor (below 10% after polymerization) in both water and propylene carbonate. Low intake of solvent might be the reason that the solvation of PPy has less effect on the actuation than the solvation of the ions that enter and are expelled during the reversible redox processes. The surface conductivity of the films (Table 2) was in range of 11 S cm^−1^ for not actuated PPy/DBS films and increased to 15–17 S cm^−1^ in most electrolyte solutions, alas the conductivity interestingly remained almost unchanged in TBACF_3_SO_3_-PC electrolyte. This effect might be related to the especially low solvation of the ions of this electrolyte.

### 3.2. Elemental Composition

**Figure 2:**
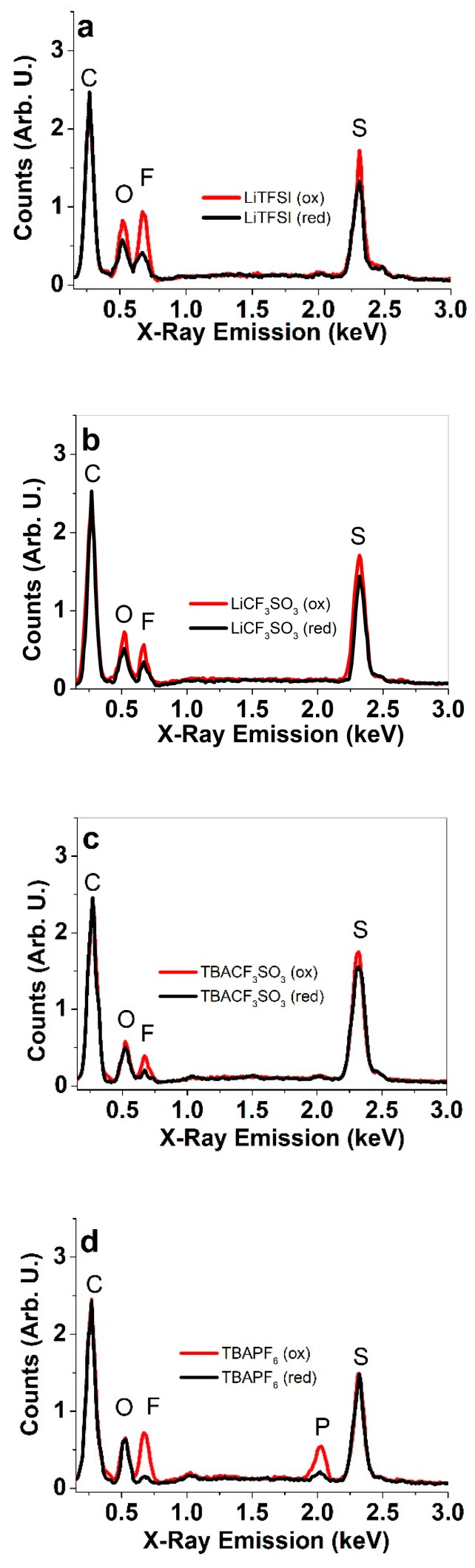
EDX spectroscopy of surface of PPy/DBS films oxidized (red line, +1.0V, 5min) and reduced (black line, -0.55 V, 5 min) in propylene carbonate solutions of (**a**) LiTFSI, (**b**) LiCF_3_SO_3_, (**c**) TBACF_3_SO_3_, and (**d**) TBAPF_6_.

To investigate the ion content of the polarized (oxidized or reduced) PPy/DBS films after medium-term actuation (200 cycles) in different electrolytes, EDX spectroscopy was performed and the results are shown in Figure 2.

**Figure 3:**
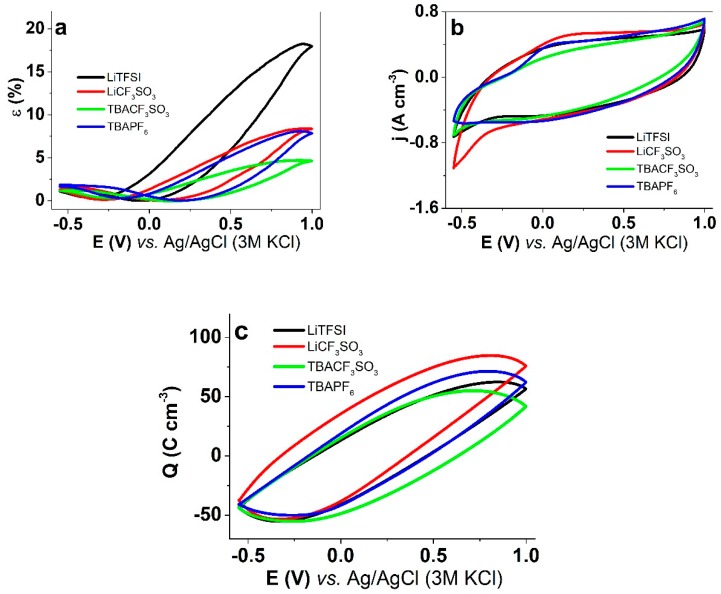
Isotonic Electro-chemo-mechanical deformation (ECMD) measurement under cyclic voltammetry (scan rate 5 mV s^−1^, 4^th^ cycle, and potential range 1V to -0.55V) of PPy/DBS films in propylene carbonate with different electrolytes: LiTFSI (black), LiCF_3_SO_3_ (red), TBACF_3_SO_3_ (green), and TBAPF_6_ (blue). The functions of potential E of (**a**) strain ε, (**b**) current density j, (**c**) charge density Q.

From the EDX results in Figure 2a–d the typical signals from carbon (C) are found at 0.27 keV, oxygen (O) at 0.52 keV, fluorine at 0.67 keV, and sulphur (S) at 2.32 keV. Figure 2 shows the relative content of elements at reduction and oxidation, the peak intensities normalized for carbon content. The content of sulphur and oxygen is partly (especially for TBAPF_6_) related to the content of the immobile DBS^−^ ions, after actuation in electrolytes with CF_3_SO_3_^−^ and TFSI^−^ anions, these anions have a larger role. The fluorine peak is present for all fluorinated anions (CF_3_SO_3_^−^, TFSI^−^, and PF_6_^−^) incorporated during the oxidation of the PPy/DBS films. Figure 3d showed an additional peak of phosphorus (P) at 2.03 keV, which occurs naturally due to the PF_6_^−^ anions. Lithium is too light of an element to be detectable. It should be observed that upon reduction, the intensities of the fluorine peaks were reduced, but in most cases (especially for LiTFSI, LiCF_3_SO_3_, and TBACF_3_SO_3_), some part of the fluorine remains detectable, indicating that some part of the anions remain trapped in the polymer film after reduction. PPy/TBACF_3_SO_3_ films have shown mixed ion actuation [20], whereas the cation activity was explained with entrapped CF_3_SO_3_^−^ anions having non-spherical shape [34], which reduces their mobility as compared to the smaller and spherical PF_6_^−^ ions [32] (agreeing well with only minor peaks of fluorine and phosphorus seen after reduction, Figure 2d). Obviously, the extent of ion flux in and out of the polymer is related to both the polarizing potential as well as the timeframe, therefore, it is also possible that to some extent the remaining anions could be expelled after longer/more intense polarization. However, for actuation, it is not desirable to overly reduce the polymer films, as the conductivity loss and possible structural collapse would significantly reduce both performance and stability.

### 3.3. Actuation and Electroactivity 

#### 3.3.1. Cyclic Voltammetry

PPy/DBS films were studied with cyclic voltammetric isotonic ECMD measurements using different electrolyte solutions in the propylene carbonate solvent. The results are shown in Figure 3.

The highest strain (Figure 3a) was seen for LiTFSI electrolyte, reaching 18% upon oxidation. In LiCF_3_SO_3_ and TBAPF_6_, a similar strain of around 8% was observed, also upon oxidation. The lowest strain was found for TBACF_3_SO_3_ with 4.7%. The existence of a small expansion at reduction (1%–1.6%, Figure 3a) present in all electrolytes, is explained by the partial entrapment of the fluorinated anions (as seen from EDX), the negative charge of which is then compensated with the ingress of cations upon reduction. The residual charge of the original dopant (DBS^−^) has clearly been compensated already during the first cycles, as especially in case of Li^+^ cations, stable (or insoluble) ion pairs have been formed that no further participate in actuation. 

The current density–time curves (Figure 3b) were overall similar, with some notable differences: (a) a more or less distinguishable oxidation wave was observed only in TBAPF_6_, which also had the least significant cation-ingress related peaks at the far negative part of the cycle; (b) in LiCF_3_SO_3_, the cation-related reduction peak was the strongest. These features agree well with the observations from the EDX spectra that the least amount of residual anions remain in the films upon reduction in case of TBAPF_6_, while those with the Li^+^ cations have the most. 

The charge density–potential curves (Figure 3c) showed close loops, identifying that in this potential range of 1.0 V to −0.55 V the systems were in so called “steady state” [35], meaning the charging/discharging was in balance and no over-oxidation or over-reduction took place. The lowest peak charge density of TBACF_3_SO_3_ is related to the lower conductivity of the films in this electrolyte (Table 2). The narrower loops, especially in case of LiTFSI, correspond to stronger electro-chemo-mechanical coupling, meaning a larger part of charge is converted to actuation. 

#### 3.3.2. Square Wave Potentials Steps

**Figure 4:**
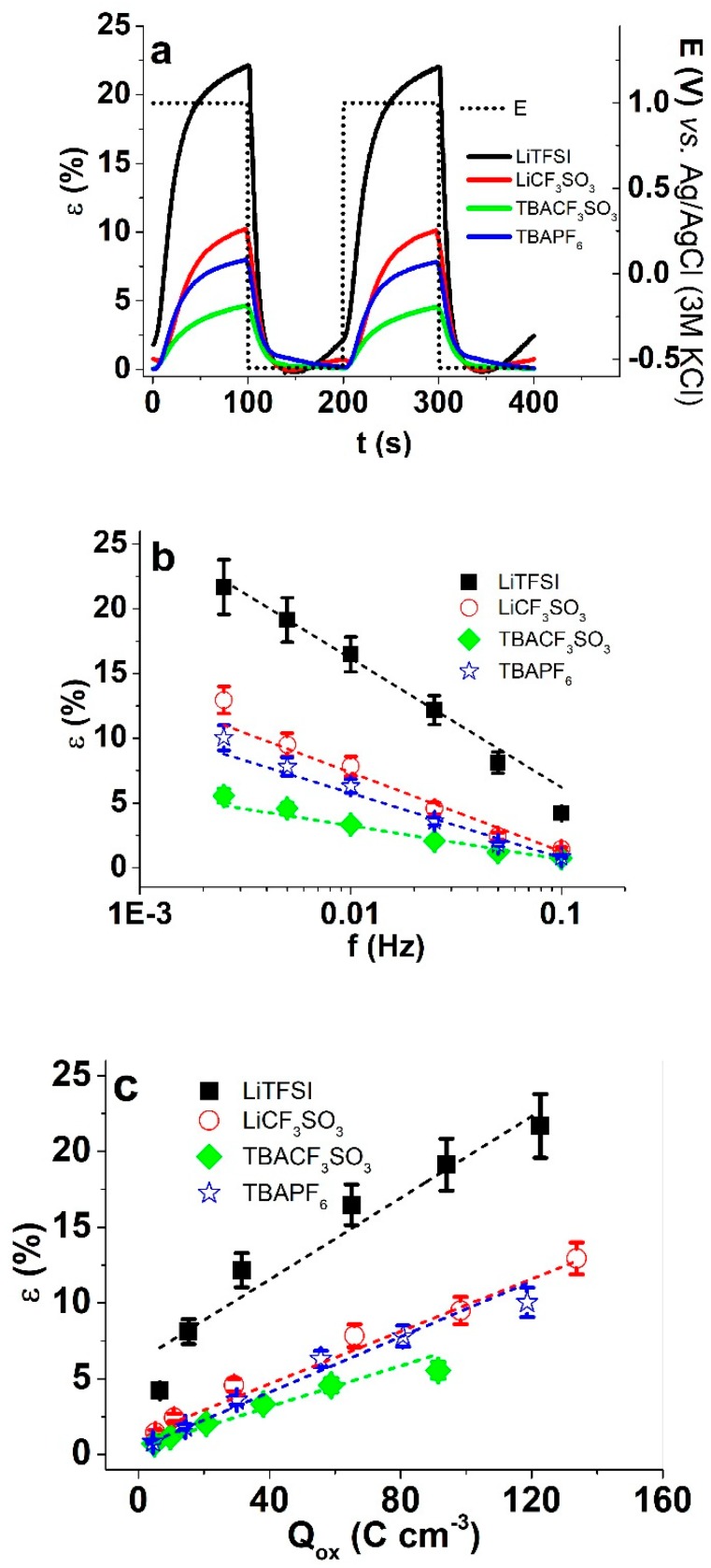
Square wave potential steps (1.0 V to -0.55 V) of PPy/DBS films in different electrolytes (LiTFSI, black, ■), LiCF_3_SO_3_ (red, ○), TBACF_3_SO_3_ (green, ♦), and TBAPF_6_ (blue, ☆) in propylene carbonate. The strain ε curves (3^rd^–5^th^ cycles, 0.005 Hz) with potential E (dashed line) of PPy/DBS films are shown in (**a**), the strain development against applied frequencies 0.0025 Hz to 0.1 Hz (logarithmic scale) in (**b**), and the strain ε against the charge density at oxidation in (**c**). The dashed lines in b and c represent the linear fit and are shown as visual guides.

From each PPy/DBS film in the four different electrolytes, three different samples were measured separately. The mean values with standard deviations are shown in Figure 4b,c.

Figure 4a shows the strain evolution of PPy/DBS films in different electrolytes. It can immediately be observed that the maximum strain of 22% upon oxidation shown by LiTFSI is one of the highest reported for PPy/DBS linear actuators. In agreement with the CV results, a small expansion upon reduction took place with strain in range of 2.5%, consequently, the strain shown in Figure 4b,c is the net strain (difference of strain at oxidation and reduction). As shown previously [36], the temperature of polymerization has an influence on the PPy/DBS film structure/packing reflected by the increased cation activity with decreasing temperature, especially below −20 °C. Therefore, while not a topic of research here, the role of cations can be further reduced by tuning the synthesis temperature. A minor cation activity was observed also for LiCF_3_SO_3_, which showed the second highest maximum strain in this study of 10%. The electrolytes with TBA^+^ as the cations showed no expansion at reduction, with purely anion-driven actuation in potential step measurements. Therefore, the driving signal shape plays an important role not only in the extent of the maximum strain, but also in the mode of actuation. The larger strain at reduction in LiTFSI than LiCF_3_SO_3_ is related to the stronger (an)ion-polymer interactions. Comparing LiCF_3_SO_3_ to LiTFSI electrolytes [37], LiTFSI is making the films both more conductive, and has a larger “plasticizing” effect, leads to a stronger reduction of elastic modulus of the PPy/DBS films (0.14 ± 0.08 MPa) in comparison to LiCF_3_SO_3_ PPy/DBS films (0.55 ± 0.04 MPa), TBACF_3_SO_3_ PPy/DBS films in range of 1.24 ± 0.1 MPa, and PPy/DBS films actuated in TBAPF_6_ electrolytes (1.12 ± 0.1 MPa). The lower elastic modulus for in LiTFSI propylene carbonate has been related to the high strain also previously [38]. However, in the case of carbon nanotubes/ionic liquid-Nafion/carbon nanotube composites [39] or other similar ionic-polymer-metal composites, the doping electrolytes have been shown to greatly influence the actuation and the blocking force [40]. In case of PEDOT:PSS/single-wall carbon nanotube composites, the strongly increased strain and stress have been attributed to the reduced elastic modulus [41], which was a function of the nature of the ionic liquid used. 

Figure 4b shows the strain as a function of frequency (0.0025 Hz to −0.1 Hz) for the four electrolytes with strain decreasing with increasing frequency. The order of maximum strain among the electrolytes matches that of the slope, indicating that stronger ion–polymer interactions (creating resistance and drag) are actually responsible for higher strain as well. Conducting polymers are Faradaic actuators [42], and the consumed charge density determines the strain, as observed from the linear dependence in Figure 4c. Importantly, the highest charge density (134 C cm^−3^) at 0.0025 Hz frequency of LiCF_3_SO_3_ did not correspond to the highest strain, which was achieved by LiTFSI with just 123 C cm^−3^. As the cations are the same for both electrolytes and the solvation of the anions is also more or less the same (and low), the interaction with the polymer matrix must be behind the difference in strain. Part of it may be the size of the anions (Table 1), which is the largest for TFSI^−^. The rest are made up by shape and specific interactions. The mobility of the ions in the polymer films can be quantitatively described by diffusion coefficients, obtained from the chronoamperometric responses [43]. Equations (5) and (6) were applied to calculate the diffusion coefficients for PPy/DBS films in different electrolytes.

(5) $$\ln\left[1-\frac{Q}{Q_{t}}\right]=-bt$$

(6) $$D=\;\frac{b*h^{2}}{2}$$

**Figure 5:**
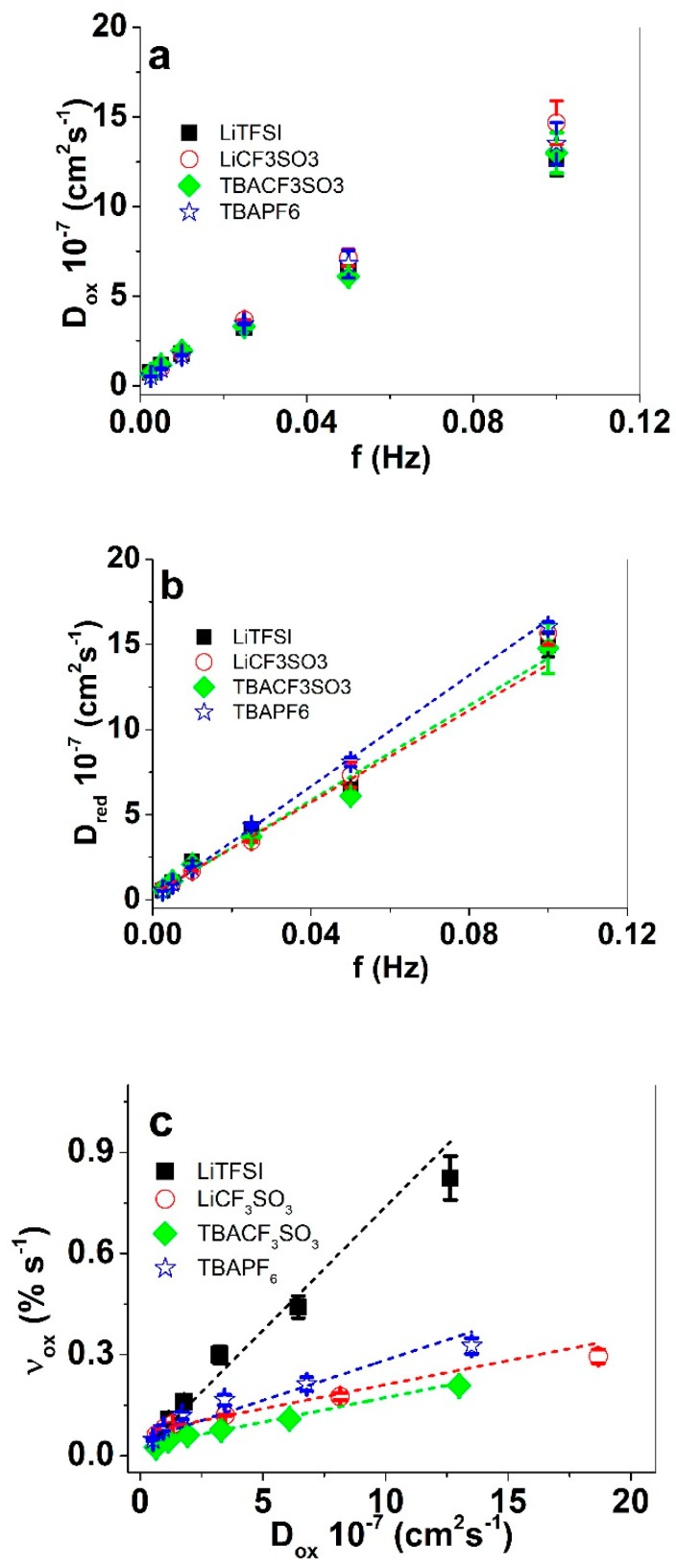
PPy/DBS films in propylene carbonate with different electrolytes (LiTFSI, black, ■), LiCF_3_SO_3_ (red, ○), TBACF_3_SO_3_ (green, ♦), and TBAPF_6_ (blue, ☆) at applied frequencies 0.0025 Hz to 0.1 Hz in potential range 1.0 V to −0.55 V. From Equations (5) and (6) the derived diffusion coefficients of PPy/DBS are shown in (**a**) at oxidation D_ox_, (**b**) at reduction D_red_ against the frequency f, and (**c**) the strain rate ν_ox_ against the diffusion coefficients at oxidation. The dashed lines in (**b**,**c**) represent the linear fits.

By integration of the current density time curves, the total charge density Q_t_ is obtained, divided by the charge density consumed by each point of time. Plotting the term on the left side of Equation (5) allows us to determine the slope b, which using Equation (6) (with thickness h of the PPy/DBS films) will give the diffusion coefficients at oxidation (Figure 5a). From the strain time curves at each applied frequency, the strain rate at oxidation was determined (Figure 5c) against the diffusion coefficient at oxidation.

The general tendency of increasing frequency leading to increasing diffusion coefficients is again demonstrated in Figure 5a,b. Lower frequencies allow the consumption of higher redox charges (oxidation and reduction time is longer), which in turn allow the certain time consuming processes such as shrinking, compaction, relaxation, and swelling of the PPy/DBS film [23] to proceed. At higher frequencies, only shrinking and swelling can take place, leading to higher diffusion coefficients. The diffusion coefficients at oxidation (Figure 5a) were rather similar for all the applied electrolytes. In case of reduction, the diffusion coefficients of TBAPF_6_ stand out from the rest as slightly higher. The smaller radius (Table 1) and the spherical shape of the PF_6_^−^ anions (Table 1) predict faster ion mobility [32]. The highest strain rate is still shown by LiTFSI (Figure 5c), due to the dominant strain in comparison to all the other electrolytes. Having mixed ion actuation, even to a small extent, has some disadvantages, especially when applied in actuation devices leading to an increase of creep during continuous cycling [20]. Therefore. electrolytes in conducting polymer actuators are favored where the actuation direction merely depends on one ion as was seen in this study for PPy/DBS linear actuators using TBAPF_6_ electrolyte, even if the strain not maximal.

## 4. Conclusions

Four different fluoride-containing salts (LiTFSI, LiCF_3_SO_3_, TBACF_3_SO_3_, and TBAPF_6_) were compared as propylene carbonate solutions for application as ionic actuator electrolytes, in our case in PPy/DBS linear actuators. With two pairs of cations, the effects of the characteristics of the three different anions are clearly distinguished. The highest strain (21.7%) (expansion upon oxidation) was shown by LiTFSI, followed by in decreasing order: LiCF_3_SO_3_ (12.7%) > TBAPF_6_ (10 %) > TBACF_3_SO_3_ (5.7%). The superior performance of LiTFSI can be explained by a combination of advantageous factors: it is the largest anion, it is non-spherical, it is known to have a plasticizing effect on PPy (lowering the elastic modulus), and overall strong interaction with the polymer network. While not the most electroactive (in terms of current or charge density), the electro-chemo-mechanical coupling was the highest, turning charge into actuation more efficiently than the other anions. On the other hand, the spherical PF_6_^−^ also stood out in several experiments, as the one with most pure anion activity (no expansion upon reduction). Interestingly, a cation effect on the otherwise anion-active films was clearly observed for the CF_3_SO_3_^−^ salts. While the cation effects depend on the driving signal as well as the time-frame and the potential window, the combination of TBA^+^ and CF_3_SO_3_^−^ cannot really be recommended for actuator applications. TBAPF_6_ should be preferred instead, as the cation role is especially low here, leading to potentially more controllable and stable actuation.

## Figures and Tables

**Table 1:** Ion radius, ionic conductivity, and solvation number in PC for ions applied in this work.

Ions	Ion Radius [nm] [29]	λ_0_ [29] [S cm^2^ mol^−1^]	Solvation Number in PC
Li^+^	0.076	8.43	3−4 [30]
TBA^+^	0.415	9.09	0 [31]
TFSI^−^	0.326	14.4	low
CF_3_SO_3_^−^	0.27	16.89	low
PF_6_^−^	0.254	17.86	low

**Table 2:** Electronic surface conductivities of PPy/DBS films.

PPy/DBS Films	Conductivity [S cm^−1^]	Thickness [µm]
After polymerization, not actuated	11 ± 1	19 ± 1.3
Actuated in LiTFSI-PC	17 ± 1.2	20 ± 1.7
Actuated in LiCF_3_SO_3_-PC	16 ± 1.1	21 ± 1.5
Actuated in TBACF_3_SO_3_-PC	10 ± 0.8	20 ± 1.1
Actuated in TBAPF_6_-PC	15 ± 1.3	21 ± 1.9

## Supplementary Materials

The following are available online at https://www.mdpi.com/2073-4360/11/5/849/s1.
