# Supplementary material for: Comparative Analysis of Fluorinated Anions for Polypyrrole Linear Actuator Electrolytes
Source: Polymers (Basel). 2019 May 10;11(5):849. doi: 10.3390/polym11050849 (PMC6571709; doi:10.3390/polym11050849)
Supplement: Supplementary file 1 [file polymers-11-00849-s001.pdf]

Supplementary

Comparative analysis of fluorinated anions for polypyrrole linear actuator electrolytes

Nguyen Quang Khuyen[1], ZaneZondaka[2], Madis Harjo[2], Janno Torop[2], Tarmo Tamm[2] and Rudolf Kiefer[1,*]

[1]Conducting polymers in composites and applications Research Group, Faculty of Applied Sciences, Ton Duc Thang University, Ho Chi Minh City, Vietnam

[2]Intelligent Materials and Systems Lab, Faculty of Science and Technology, University of Tartu, Nooruse 1, 50411 Tartu, Estonia

*Corresponding author. Tel: +886 905605515. E-mail: rudolf.kiefer@tdtu.edu.vn (Rudolf Kiefer)

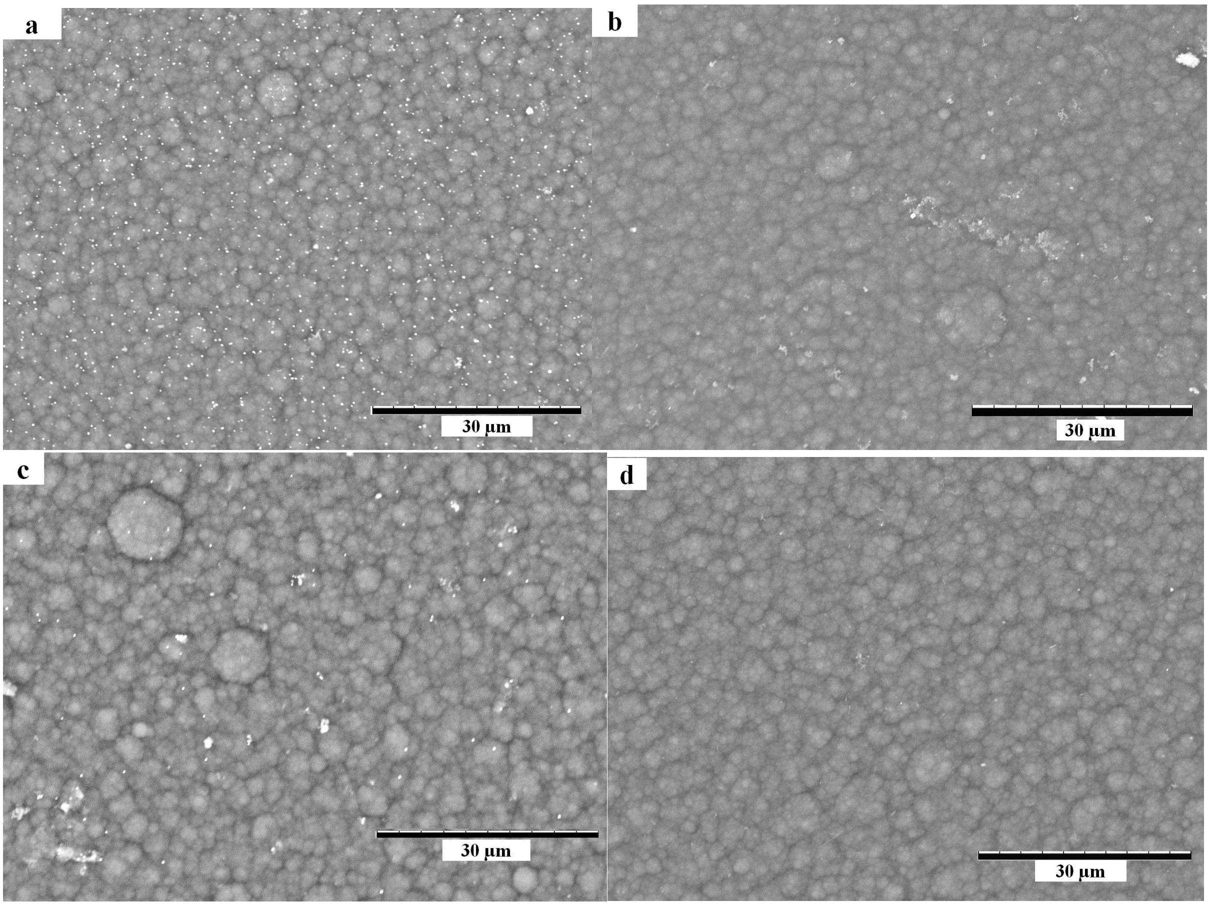

Figure S1: The SEM surface images at oxidation state (+1.0 V) (scale bar 30 µm) are shown for PPy/DBS films actuated in in a: LiTFSI, b: $LiCF_3SO_3$, c: $TBACF_3SO_3$ and d: $TBAPF_6$.
